# Satisfaction survey with DNA cards method to collect genetic samples for pharmacogenetics studies

**DOI:** 10.1186/1471-2350-7-45

**Published:** 2006-05-08

**Authors:** Jose M Vidal-Taboada, Mercedes Cucala, Sergio Mas Herrero, Amalia Lafuente, Albert Cobos

**Affiliations:** 1 Pharmacogenomic Unit, RDES SL, Barcelona, Spain; 2 Department of R&D, Novartis Farmacéutica SA, Barcelona, Spain; 3 Department of Pharmacology and Clinical Therapeutics, Faculty of Medicine, University of Barcelona, Barcelona, Spain; 4 Genetic Unit. Department of Physiological Sciences I. Faculty of Medicine, University of Barcelona, Barcelona, Spain

## Abstract

**Background:**

Pharmacogenetic studies are essential in understanding the interindividual variability of drug responses. DNA sample collection for genotyping is a critical step in genetic studies. A method using dried blood samples from finger-puncture, collected on DNA-cards, has been described as an alternative to the usual venepuncture technique. The purpose of this study is to evaluate the implementation of the DNA cards method in a multicentre clinical trial, and to assess the degree of investigators' satisfaction and the acceptance of the patients perceived by the investigators.

**Methods:**

Blood samples were collected on DNA-cards. The quality and quantity of DNA recovered were analyzed. Investigators were questioned regarding their general interest, previous experience, safety issues, preferences and perceived patient satisfaction.

**Results:**

151 patients' blood samples were collected. Genotyping of *GST *polymorphisms was achieved in all samples (100%). 28 investigators completed the survey. Investigators perceived patient satisfaction as very good (60.7%) or good (39.3%), without reluctance to finger puncture. Investigators preferred this method, which was considered safer and better than the usual methods. All investigators would recommend using it in future genetic studies.

**Conclusion:**

Within the clinical trial setting, the DNA-cards method was very well accepted by investigators and patients (in perception of investigators), and was preferred to conventional methods due to its ease of use and safety.

## Background

Pharmacogenetic studies are essential both for understanding the variability of drug response observed among patients and furthering the development of personalized medicine [1-3]. Peripheral blood sample collection from patients is a critical step in obtaining DNA for genetic or pharmacogenetic studies. This may require the involvement of different centres, couriers and laboratory resources. A lack of laboratory resources or specialized personnel can restrict the participation of some medical centres [4,5].

Blood samples obtained by venepuncture (the standard DNA collection method) often cannot be used due to medical, logistical or cultural reasons. Occasionally, use of such samples is feasible, but prohibitively expensive. This method is a labour-intensive way of obtaining DNA at the doctor's office and is associated with potential security problems. The sample must either be delivered rapidly to the central laboratory or frozen at the doctor's office. Logistical difficulties in managing liquid or frozen samples are associated with high costs [6, 7].

Recently, a new method has been described that is easier to use, less invasive and much cheaper than the usual venepuncture technique. This new method uses chemically treated paper cards, such as IsoCode Cards^® ^or FTA Cards^® ^(also known as DNA Cards). The DNA cards method allows dried blood samples (obtained from fingertip puncture) to be stored and transported at room temperature [6,7]. High-quality genomic DNA can be extracted from these samples and used for genetic analysis, providing suitable archival media in DNA banks for forensic [8] or genetic epidemiological studies [4].

Several authors have compared traditional methods and the DNA cards method for collection of DNA samples indicating some advantages of the blood spots and DNA cards method for multicenter studies [7-9]. The DNA cards method has been used to collect DNA samples in several clinical studies focusing on infective pathogens, such as malaria [5,10], staphylococcus [11], or HIV [12], Gaucher disease [13] and cancer [4,14]. This method was selected to perform a pharmacogenetic substudy. DNA cards were used to obtain blood samples in a multicentre clinical trial (ITEMS) in which the response to tegaserod (Zelnorm^®^/Zelmac^®^, Novartis Pharmaceuticals AG), a selective 5-HT_4 _receptor partial agonist, was evaluated in patients with irritable bowel syndrome. The aims of the present study were to evaluate the implementation of the DNA cards method in a clinical trial, the quality and quantity of DNA collected from patients, the investigators' satisfaction and their perception of the patients' satisfaction with this method.

## Methods

### Study design and subjects

Patients from the ITEMS study [15] provided written, informed consent to participate in the pharmacogenetic substudy. The study protocol was approved by the Institutional Review Boards of the 24 participating centres and the Spanish Health Authorities. The ITEMS study personnel in these centres (hospital and primary care) were involved in the collection procedure and the satisfaction survey.

### Sample collection using the DNA cards method

**Figure 1:**
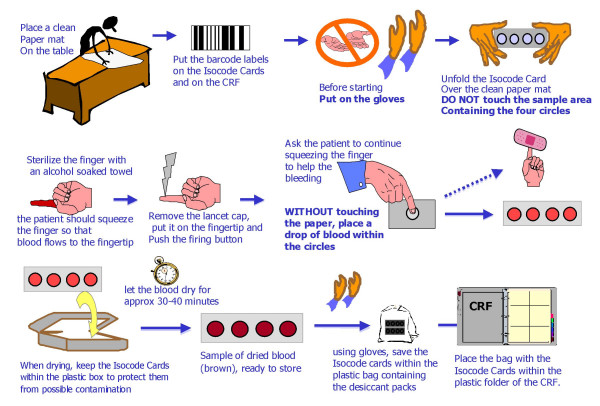
Sample blood collection diagram at doctor's office using the DNA cards method.

Each investigator received a blood sample collection kit, comprising a plastic container with two DNA card devices (IsoCode Cards^®^, Schleicher & Schuell/Whatman), an automatic lancet, an alcohol-soaked towel, a Band-aid^®^, two desiccant packs, a zip-sealed plastic bag, an instructions card and patient identification barcode labels. Patient blood samples were obtained according to the procedure shown in Figure 1. Several drops of blood from a fingertip puncture were collected on each IsoCode Card^® ^and dried at room temperature for 30–40 minutes to stabilize the sample. The IsoCode Cards^® ^were then stored at room temperature alongside the desiccant packs (to avoid humidity) in the zip-sealed plastic bag, before being sent (at the investigator's convenience) by standard courier to the DNA bank for DNA purification (1–8 months after blood collection).

### DNA isolation and quantification

DNA was extracted using a modification of the manufacturer's instructions. Using a paper puncher, 1/8 inch (3.2 mm) discs were punched from the complete card matrix region containing the dried blood. Between consecutive uses, the puncher was sterilized with alcohol and flame before making several punches through clean filter paper. The discs containing dried blood were placed in a 1.5 mL tube and heated in an oven at 80°C for 15–20 minutes. To remove contaminants, the discs were washed twice with 500–750 μl of water and vortexed three times for 5 seconds. The washed disc was transferred to a new tube and 12 μL of water was added per punch. The individual tubes were incubated at 100°C for 15 minutes to elute the DNA from the card matrix, before being stored on ice. The samples were pulse-vortexed about 60 times and centrifuged for 1 minute at 13,000 x g. The matrix discs were removed using plastic forceps, and squeezed on the side of the tube to remove excess water. The eluate containing the purified single-stranded DNA (ssDNA) was quantified and stored at -80°C until required. ssDNA yields were determined as instructed in the Oligreen ssDNA quantitation kit (Molecular Probes) using a spectro-fluorometer (Hitachi F-4500).

### Genotyping quality control

The *GSTM1 *and *GSTT1 *polymorphisms were assayed using a multiplex PCR based on a previously described method [16]. *GSTM1 *(5'-GTG CCC TAC TTG ATT GAT GGG-3', 5'-CTG GAT TGT AGC AGA TCA TGC-3') and *GSTT1 *(5'-TTC CTT ACT GGT CCT CAC ATC TC-3', 5'-TCA CCG GAT CAT GGC CAG CA-3') [17] primers were used to amplify a 273 and 459 base-pair (bp) fragment, respectively. The absence of both PCR products indicated a deleted genotype. A 349 bp fragment of the *human actin *gene was included as an internal PCR control (primers 5'-GGG CAC GAA GGC TCA TCA TTC-3', 5'-TTT TTG GCT TGA CTC AGG ATT-3'). Length specific PCR amplifications of the primer sets used were confirmed by an in silico search of the Genome sequence using the UCSC In-Silico PCR software [18, 19]. PCR reactions were conducted in a 50 μL volume at standard condition with 1.5 mM MgCl_2_, 4 μg/mL of each primer, 2.5 units of Amplitaq (Applied Biosystems) and 25 ng of gDNA. The PCR cycling conditions were: 5 minutes at 95°C followed by 95°C for 10 seconds, 61°C for 30 seconds and 72°C for 30 seconds during 30 cycles, with a final extension period of 10 minutes at 72°C, performed in a Perkin Elmer 2400 cycler. Genotypes were identified by electrophoresis of the amplified fragments through 2% agarose gels containing ethidium bromide (0.5 mg/mL).

### Satisfaction survey

Twenty-eight investigators (17 principal investigators, 5 co-investigators and 6 nurses) from 17 hospital outpatient clinics and seven primary care centres were surveyed regarding the DNA cards method. Questions focused on general interest in pharmacogenetic studies, previous experience of collecting blood samples, safety, preferred methods of blood collection and perceived patient satisfaction with the DNA cards method used.

### Statistical analyses

All statistical analyses were performed using the SAS System^® ^version 8.02 software (SAS Institute, Inc). Data were summarized as mean (standard deviation [SD]) or n (%) unless otherwise stated. Non-parametric procedures (Kruskal-Wallis, Wilcoxon rank sum or Spearman correlation test, as appropriate) were used to investigate a potential investigator effect, as well as the possible influence of patient characteristics (gender, age, education level) on the number of discs obtained from the DNA cards. Multiple regression was used to build a predictive model of DNA recovered from DNA cards. The significance of regressors was based on Wald statistics.

## Results

### Blood collection and DNA analysis

Blood samples were obtained from 151 patients by 28 investigators using the DNA cards method. Four investigators reported previous experience of collecting samples for genetic analysis. Samples were obtained at the doctor's office and transported by courier to the DNA bank. Samples were stored at room temperature until use, with a variable storage period ranging from 5 to 498 days (median 248 days). Deviations in the collection procedure were observed in 62 of the 151 samples (41%) received by the DNA bank. These included incorrect sealing of the bag (n = 34, 55%), failure to include desiccant packs (n = 6, 10%), blood expanding out of the card collection region (n = 15, 24%), cards arriving with no identification label attached (n = 2, 3%), and others (n = 5, 8%). A small amount of blood on the paper area (fewer than 17 punches or less than 30% of total area) was observed in nine samples (6%).

The area of dried blood on the matrix card varied considerably between samples. While some patients stained the specimen cards completely, others stained only a small portion of the cards. We obtained a range of 11 to 55 punched discs (1/8 inch) per patient, with a mean of 34.6 (10.0) discs (Table 1). The number of punched disc obtained was heterogeneous among investigators (Kruskall-Wallis test, p < 0.0001). None of the patients' characteristics was associated with the number of discs obtained from IsoCode Cards^®^.

**Table 1:** Frequency distribution of amounts of total DNA extracted, DNA concentration by punch and punch by DNA cards received at the DNA bank

**Total ssDNA (ng)**	**Samples (n)**	**%**	**Accumulative %**
30–250	18	11.84	11.84
251–1000	59	38.82	50.66
1001–2000	52	34.21	84.87
2001–3000	21	13.82	98.68
>3000	2	1.32	100.00
**ng ssDNA/punch**			
1–10	22	14.47	14.47
11–20	33	21.71	36.18
21–30	26	17.11	53.29
31–40	28	18.42	71.71
41–50	22	14.47	86.18
51–60	16	10.53	96.71
>60	5	3.29	100.00
**Punch/card**			
11–20	11	7.24	7.24
21–30	31	20.39	27.63
31–40	48	31.58	59.21
41–50	52	34.21	93.42
>50	10	6.58	100.00

DNA purification of the punched discs recovered 30–4983 ng of ssDNA, with a mean of 1097.9 (± 789.7) ng and a median of 962 ng. The yields of DNA per disc were variable (range, 1.7–115.9 ng/disc) with a mean of 29.6 (± 18.1) ng of ssDNA per disc and a median of 26.5 ng. Table 1 shows the frequency distribution of the extracted quantities of DNA. The best-fit regression model of the log-transformed amount of DNA (ng) accounted for 65% of the total variance, including linear and quadratic terms for the number of discs (p = 0.001 in both cases) and a linear term for storage time (p = 0.019). The regression equation obtained was:

ln [DNA] = 0.3038*p - 0.0034*p^2 ^+ 0.0018 t

where (p) is the number of punched discs and (t) is the storage time expressed in days.

**Figure 2:**
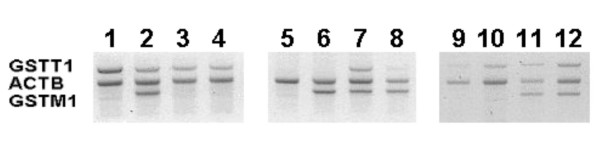
**Example of GST genotypes obtained from blood samples collected using the DNA cards method**. Samples received with procedure deviations (lanes 1–4), collected according to the study procedure (lanes 5–8), and samples with low DNA recovering (lanes 9–12).

The quality of purified DNA was assessed by genotyping the *GST *gene. Positive PCR amplifications were obtained from all patient samples and their corresponding *GST *genotypes at the first attempt (except in one case where a second PCR analysis was performed) resulting in a 100% success rate according to the presence/absence of the length specific PCR products (Figure 2). The following genotypes were identified: 82 patients (54.0%, 95%CI: 45.7–62.1%) had the GSTM1*0 genotype and 20 patients (13.2%, 95%CI: 8.1–19.4%) had the GSTT1*0 genotype. When analyzing the two loci simultaneously, 12 patients (8%, 95%CI: 4.2–13.5%) had the combination GSTM1*0/T1*0 genotype, and 63 patients (41.7%, 95%CI: 33.7–50.0%) had the combination *GSTM1*/*T1*.

No problems such as cross contamination or DNA degradation occurred during the purification and genotyping process. There were no significant differences in the quality and quantity of DNA recovered between specimens collected correctly and specimens showing some of the protocol violations reported earlier (Figure 2).

### Satisfaction survey

**Figure 3:**
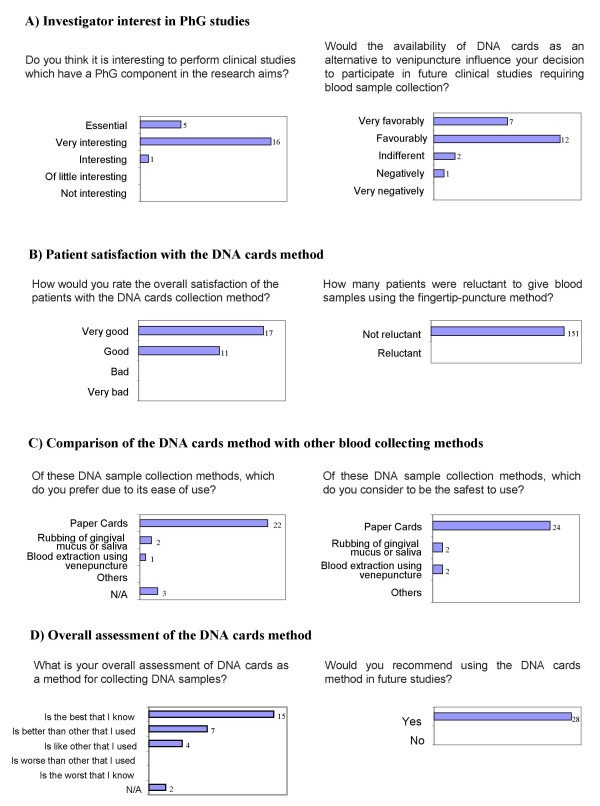
Satisfaction survey with DNA cards method.

Investigators from 22 of the 24 participating centres completed the survey. All 22 principal investigators and co-investigators surveyed believed that it was interesting to perform research studies that had a pharmacogenetic component (Figure 3a). Most principal investigators (86.4%) declared that, compared with venepuncture, use of the DNA cards method would 'favourably' or 'very favourably' influence their decision to participate in future pharmacogenetic studies (Figure 3a).

Patient satisfaction with the DNA cards method (as perceived by the 28 principal investigators, co-investigators or nurses) was either 'very good' (n = 17, 60.7%) or 'good' (n = 11, 39.3%). There was no reported resistance from patients to the fingertip-puncture technique (Figure 3b). Only five investigators reported problems obtaining sufficient blood, all of which were associated with the fingertip-puncture process (difficulties with the lancet) or with the application of blood onto the paper card.

When comparing the DNA cards method with other DNA collection techniques (such as venepuncture or saliva sampling), most investigators (n = 22, 78.6%) preferred the DNA cards method due to ease of use. The DNA cards method was also considered the safest blood-collecting method (Figure 3c).

When asked for an overall assessment of the DNA cards method, the majority of investigators (n = 22, 78.6%) declared that the DNA cards method was the best option for collecting DNA compared with other usual methods. All 28 investigators indicated that they would recommend using DNA cards for obtaining DNA samples in future genetic studies (Figure 3d).

## Discussion

We evaluated a DNA collection method that used DNA cards in the context of a clinical trial with a pharmacogenetic substudy. We also assessed the impact of this method on investigator satisfaction and their patients' perceived satisfaction.

The results of this survey demonstrate a high level of investigator satisfaction with the DNA cards method. These findings corroborate data reported in other studies using this methodology, which have focused on the views and perceptions of the investigators [5,7]. DNA cards have many advantages. When collecting blood, the investigators and patients stated their acceptance of, and preference for, the DNA cards method compared with other collection techniques. From the investigators' perspective, the DNA cards collection method using fingertip-puncture is easily and quickly administered, which decreases the staff's exposure to blood, therefore making this method safer than other blood collection techniques. Additionally, the cards are easy to label and handle, do not require preprocessing at the doctor's office or cool storage (which would require appropriate freezers at the centre). From the patients' perspective, this method could be perceived as minimally invasive, painless and easy to perform compared with venepuncture collection.

The results of our model indicate that the storage period has a slightly favourable influence on the recovery of DNA from the cards, with an additional effect during the first 6 months (data not shown). These results are consistent with previous studies, which reported very good DNA stability in dried blood samples on DNA cards stored at room temperature for up to 16 months [7,8]. Other authors have reported successful PCR analyses after 4 or 8.5 years of storage [20,21] and the manufacturer asserts that it is possible to obtain PCR amplifications after 14 years of DNA storage (Whatman brochure). This stability provides a great advantage over other methods, as it simplifies trial logistics, management and costs. Transport, storage, and DNA extraction can all be performed at room temperature, making it easier to transport the samples from the various collection centres to the central DNA bank or laboratory. This drastically reduces the work necessary for sample management, as well as lowering the costs of the process for several reasons: fingertip puncture is less labour intensive than venepuncture ; freezer or refrigerator management and handling of frozen samples is not needed, and low-temperature packaging and specialized couriers are not required. Additionally, the duration of storage after blood collection (before the sample can be transported) is much longer than is possible with fresh blood samples, allowing several samples to be transported together, thus reducing both the total number of parcels transported and the associated control management. This increased storage time also removes time constraints on receipt of samples at the central laboratory, as blood samples do not require immediate processing after delivery to the laboratory. In addition, transport by standard mail or courier is inexpensive (cards are compact and lightweight), the cards are economical and require little storage space [22], and the working time required for sample management by the investigators and study monitors is shorter than with conventional methods based on liquid or frozen sample handling. Based on our experience, we estimated that using DNA Cards predicted a 56% saving in samples collection and management (material and reagent cost, freezers, control and sample management, transport of specimens and purification of DNA) as compared to the alternative use of frozen samples. Consequently, DNA cards were perceived as a cost-minimising method to perform genetics analyses, as other authors have pointed [6,22].

There are traditional methods such as the use of buccal mucus sampling that are non invasive alternative to blood collection. Mouthwash allows to obtain genomic DNA of high molecular weight from expectorations, an easy way of sample collection for adults but not an option for infants and small children where citobrush are required. This method has been used in several large epidemiologic studies showing that DNA obtained from buccal cells allows PCR amplification but the yields of DNA are highly variable between specimens and bacterial contamination must be addressed [9]. The stability of the DNA is good during 5 days allowing samples to be transported in fresh. In order to increase the stability in time of the DNA from buccal cells, it has been reported the use of DNA cards to store this DNA during years at room temperature. Specific formats of DNA cards have been developed including an indicator to check the correct collection of buccal cells over the modified matrix of the cards. The major advantage of the collection of saliva in DNA cards is that is a non-invasive method compared to fingerpunction, but the disadvantage is that yield of DNA recovered from saliva specimens are more variable than blood specimens and sometimes is necessary to collect again samples of subjects which have shown a low recovery of DNA from their specimens [6,7,22]. Also there is the possibility of coexistence of different genomes in the mouth, that could interfere in some cases in the correct assignation of the genotype of the subject.

The DNA obtained in this study was of sufficiently high quality to perform PCR analysis after 16 months of storage. Overall, the success rate for the PCR genotyping of the *GST *gene was 100% according to the presence/absence of the corresponding length specific PCR products. We have only observed a problem with a single sample in our study that could have been related to the low yield of DNA from that particular sample. The method is robust for PCR analysis and the protocol violations observed (such as incorrect sealing of the bag and failure to include desiccant packs) did not affect the quantity or quality of the purified DNA. The frequencies of the *GSTM1 *(13.2 %) and *GSTT1 *(53.9%) genotypes are similar to those reported in other studies of Caucasian populations from Spain [23] and the differences are not statistically significant. Genotyping analysis of the SNP rs2281820 locus (corresponding to the motilin gene) in a subset of 140 samples showed that genotype frequencies are in Hardy-Weinberg equilibrium (data not shown) indicating that DNA samples collected by this method do not generate methodological or sampling problems for genotyping. At present, the method is evaluated for other genotyping techniques, such as minisequencing (CYP2C9) or Taqman [24], without problems until now (data not shown).

The main disadvantage of the DNA cards method is the quantity of DNA recovered compared with the yields obtained with fresh venous blood samples. The mean of 1043 ng (median = 901 ng) of purified DNA per patient that we obtained from two paper cards was considerably less than the usual quantities (5–10 μg) obtained from the purification of 0.3 mL of fresh or frozen whole blood by affinity columns or cell lysis and protein precipitation methods [25]. This observation could be explained partly by the fact that 40% of the DNA is retained on the card matrix [6]. In addition, the amount of DNA is highly variable between patient specimens, due to intrinsic factors such as the proportion of white blood cells in the blood [7] and, primarily, to the quantity of blood actually deposited on the card matrix (as shown in our model). As reported here, this last factor is influenced by the investigator, who should be aware of the importance of completely staining the sample collection area of the card. It is, therefore, important that study monitors instruct the investigators of the need to stain the entire sample card with the patient's blood, to maximize the quantity of DNA recovered.

The amount of DNA purified from the DNA cards could be problematic for use in molecular analyses requiring quantities of micrograms, such as Southern blot or genomic cloning, but is suitable for use in SNP analyses. The amount of DNA obtained potentially allows dozens to hundreds of polymorphisms to be analysed using multiplexed PCR analyses. This capacity for analysis of polymorphisms is usually sufficient to achieve the objectives of pharmacogenetic studies, if the genes involved in the pharmacokinetic and pharmacodynamic pathways are known. If more polymorphism analyses are required, or in the case of recovering lower quantities of DNA from patients, investigators will need to use any one of the whole genome amplification methods that have been published, such as multiple displacement amplification [26], to obtain a sufficient quantity of genomic DNA for analysis. Epigenetic modifications have been described to be an important mechanism for gene regulation and disease [27]. At present only one paper has reported the use of DNA cards to perform methylation studies [28]. If DNA Cards have to be used for analyses of genomic methylation which requires DNA chemical modification, such as methylation specific PCR (MSP) technique, it may be necessary to carry out a previous study to validate the use of DNA cards for these techniques. 

In the satisfaction survey, all investigators would recommend using the DNA cards method in future genetic studies, due to its ease of use and its safety. The principal investigators from the centres surveyed showed interest in carrying out further pharmacogenetic studies, indicating that the availability of the DNA cards method would favourably influence their decision to participate in similar studies in the future. Based on investigator satisfaction, the DNA cards technique could be the method of choice for collecting DNA samples in phase III or IV clinical trials evaluating the genetic influence of several (1–100) polymorphisms in patients' response to a pharmacological treatment. Clinical trials typically involve multiple centres, sometimes with varying levels of laboratory resources, DNA cards are thus a logical and practical choice.

## Conclusion

Within a clinical trial setting, the DNA-cards method was very well accepted by investigators and patients (in perception of investigators), and was preferred to conventional methods due to its ease of use and safety. This method is very robust allowing to obtain DNA of quality to perform PCR analyses and obtain the GSTM1 and GSTT1 genotype of all patients of the study. Overall, the DNA-cards method is a tool that facilitates genetic and pharmacogenetic testing in the usual clinical practice at doctor's office.

## Competing interests

JMV has been investigator and project Manager, and AC technical director, at the Pharmacogenomic Unit of RDES, a CRO (Contract Research Organization), working in the design, management and analysis of pharmacogenetics and clinical studies. JMV and AC declare that don't have maintained agreements or other economical interest with any DNA cards manufacturers, except the usual purchase of the devices at supplier's market prices. The other author(s) declare that they have no competing interests.

## Authors' contributions

JMV participated conceiving, designing and coordinating the whole study, participated in the sample/data management, the statistical analyses and interpretation of results and drafted the manuscript. MC participated in the coordination, acquisition of funding, conceiving and design of the survey and helped in drafting the manuscript. SM and AL participated designing and carrying out the molecular genetic analyses and revised critically the draft of the manuscript. AC participated in the design of the whole study, performing the statistical analyses, interpreting the results and helped in drafting the manuscript. All authors read and approved the final manuscript.

## Pre-publication history

The pre-publication history for this paper can be accessed here:


